# Mismatch Repair Deficiency as a Predictive and Prognostic Biomarker in Molecularly Classified Endometrial Carcinoma

**DOI:** 10.3390/cancers13133124

**Published:** 2021-06-22

**Authors:** Mikko Loukovaara, Annukka Pasanen, Ralf Bützow

**Affiliations:** 1 Department of Obstetrics and Gynecology, Helsinki University Hospital, University of Helsinki, 00290 Helsinki, Finland; ralf.butzow@hus.fi; 2 Research Program in Applied Tumor Genomics, Department of Pathology, Faculty of Medicine, Helsinki University Hospital, University of Helsinki, 00290 Helsinki, Finland; annukka.pasanen@hus.fi

**Keywords:** endometrial cancer, mismatch repair, *polymerase*-*ϵ*, p53, The Cancer Genome Atlas

## Abstract

**Simple Summary:**

We studied mismatch repair (MMR) deficiency as a predictive and prognostic biomarker in endometrial carcinoma. MMR deficiency was associated with poor outcome only when p53 aberrant and *polymerase*-*ϵ* mutant tumors were excluded from the MMR proficient subgroup, in accordance with molecular classification based on The Cancer Genome Atlas. MMR deficiency was associated with an increased risk of death in the absence of various clinicopathologic risk factors, but the outcome was not worsened when such risk factors were present. The proportion of pelvic relapses and lymphatic dissemination, defined as primary lymph node involvement or relapses in regional lymph nodes, were higher in the MMR deficient subgroup. In conclusion, the effect of MMR deficiency on the outcome of endometrial carcinoma depends on how MMR proficiency is defined. MMR deficiency is associated with an increased risk of death in the absence of established risk factors and a unique pattern of disease spread.

**Abstract:**

The aggressiveness of mismatch repair (MMR) deficient endometrial carcinomas was examined in a single institution retrospective study. Outcomes were similar for MMR proficient (*n* = 508) and deficient (*n* = 287) carcinomas, identified by immunohistochemistry. In accordance with molecular classification based on The Cancer Genome Atlas (TCGA), tumors with abnormal p53 staining or *polymerase*-*ϵ* exonuclease domain mutation were excluded from the MMR proficient subgroup, termed as “no specific molecular profile” (NSMP). Compared with NSMP (*n* = 218), MMR deficiency (*n* = 191) was associated with poor disease-specific survival (*p* = 0.001). MMR deficiency was associated with an increased risk of cancer-related death when controlling for confounders (hazard ratio 2.0). In the absence of established clinicopathologic risk factors, MMR deficiency was invariably associated with an increased risk of cancer-related death in univariable analyses (hazard ratios ≥ 2.0). In contrast, outcomes for MMR deficient and NSMP subgroups did not differ when risk factors were present. Lymphatic dissemination was more common (*p* = 0.008) and the proportion of pelvic relapses was higher (*p* = 0.029) in the MMR deficient subgroup. Our findings emphasize the need for improved triage to adjuvant therapy and new therapeutic approaches in MMR deficient endometrial carcinomas.

## 1. Introduction

About 30% of endometrial carcinomas exhibit a defect in the DNA mismatch repair (MMR) pathway [1]. MMR deficiency contributes to microsatellite instability (MSI), which is characterized by a high level of gene mutations [2]. MMR deficient endometrial carcinomas are mostly sporadic, resulting from hypermethylation of the *MLH1* promoter or less frequently from silencing of the other MMR genes MSH2, MSH6, or PMS2. About 3% of patients have an inherited mutation in one or more MMR genes (Lynch syndrome) [3]. Universal tumor testing for MMR deficiency, either by MMR protein immunohistochemistry or microsatellite instability (MSI) measurement, is recommended for the screening of Lynch syndrome and potential responders to immunotherapy [4].

MMR proteins have been extensively studied as predictive and prognostic biomarkers of endometrial carcinoma. MMR deficiency is reported to predict the presence of high-risk features of the disease, including old age, advanced stage, and uterine risk factors [5,6,7,8,9,10]. However, no consistent association between MMR and poor outcome has been found, although three studies have reported an association with either poor recurrence-free survival [7,11] or progression-free survival [8] in univariable analyses. We found that *MLH1* methylated carcinomas predict diminished disease-specific survival even after controlling for confounders [9].

MSI is a characteristic signature of one of the four molecular subgroups of endometrial carcinoma described by The Cancer Genome Atlas (TCGA) [12]. To further elucidate the role of the MMR system in determining the aggressiveness of endometrial carcinoma, we studied MMR proteins as predictive and prognostic biomarkers in a cohort that was classified into molecular subgroups based on TCGA.

## 2. Materials and Methods

### 2.1. Study Population and Data Collection

This was a retrospective study of patients who underwent surgical treatment for stage I–IV endometrial carcinoma at the Department of Obstetrics and Gynecology, Helsinki University Hospital, between 1 January 2007 and 31 December 2012. Clinicopathologic data were extracted from institutional medical and pathology records. Stage was determined according to the International Federation of Gynecology and Obstetrics guidelines revised in 2009 [13].

Disease-specific survival was calculated as the time from surgery to death from endometrial carcinoma. Cause of death was mainly based on medical records. Missing data were complemented from death certificates derived from Statistics Finland.

The following variables were controlled for as confounders in survival analyses:

(i) age [14];
(ii) stage [15];
(iii) uterine risk factors (depth of myometrial invasion, cervical stromal invasion, tumor size, lymphovascular space invasion) [16,17,18];
(iv) peritoneal cytology finding [19,20,21,22];
(v) L1 cell adhesion molecule (L1CAM) expression [23,24,25,26].

The cutoff for age was set at 65 because being over 65 years of age is a poor prognostic factor in endometrial carcinoma [14]. The choice of 5 cm as a determinant for the analysis of tumor size was based on the finding that size approximating the entire uterine cavity is strongly associated with survival in stage I endometrial carcinoma [27]. Lymphovascular space invasion was defined as the presence of adenocarcinoma, of any extent, in endothelium-lined channels of uterine specimens outside the tumor. Peritoneal cytology was considered positive if adenocarcinoma cells were detected from the peritoneal washes obtained during surgery, regardless of the number of cancer cells.

Standard surgery included total hysterectomy and bilateral salpingo-oophorectomy. Lymphadenectomy was performed in selected patients. Adjuvant therapy decision was based on stage and histologic findings at surgery. Patients with early stage endometrioid carcinoma with high-risk features generally received either vaginal brachytherapy or whole pelvic radiotherapy. Vaginal brachytherapy was preferred in patients who underwent surgical nodal assessment. Patients with nonendometrioid or advanced-stage endometrioid carcinoma were treated with combined chemotherapy and radiotherapy. Paclitaxel/carboplatin doublet was the adjuvant chemotherapy of choice. The study followed the reporting recommendation of tumor marker studies (REMARK) guidelines [28].

### 2.2. Molecular Classification

Tumors were categorized into molecular subgroups according to a modified TransPORTEC classifier that recapitulates the four subgroups of the TCGA as follows: (1) mismatch repair deficient (MMR-D, surrogate to microsatellite instability hypermutated in the TCGA classification system); (2) p53 abnormal (p53 abn, surrogate to copy-number high); (3) *polymerase*-*ϵ* (*POLE*) ultramutated; and (4) “no specific molecular profile” (NSMP, surrogate to copy-number low) [29,30]. A tissue microarray was constructed on primary tumor samples as previously described [26]. The following monoclonal antibodies were used for chromogenic immunohistochemistry: MLH1 (ES05, Dako, Santa Clara, CA, USA); MSH2 (G219-1129, BD Biosciences, San Jose, CA, USA); MSH6 (EPR3945, Abcam, Cambridge, UK); PMS2 (EPR3947, Epitomics, Burlingame, CA, USA); p53 (DO-7, Dako); and L1CAM antibody clone 14.10 (SIG-3911, Covance, Princeton, NJ, USA). Tissue microarray slides were scanned with a three-dimensional Histech Pannoramic 250 Flash II scanner by Fimmic Oy (Helsinki, Finland). Slide images were managed and analyzed with WebMicroscope Software (Fimmic Oy). Virtual slides were scored by a pathologist blinded to clinical data. Equivocal cases were examined by a second investigator and a consensus was reached. MMR status was considered deficient when a complete loss of nuclear expression in carcinoma cells of one or more MMR proteins (MLH1, MSH2, MSH6, PMS2) was detected by immunohistochemistry. Aberrant p53 staining was defined as strong and diffuse nuclear staining or completely negative (“null”) staining in carcinoma cells. Weak and heterogeneous staining was classified as wild-type expression. Stromal and inflammatory cells served as internal controls for MMR and p53 staining. A membranous staining of ≥10% was considered positive for L1CAM expression [26]. *POLE* exonuclease domain mutation (EDM) screening of hot spots in exons 9, 13, and 14 was performed by direct sequencing [31]. Only samples with high-quality sequence for all four examined *POLE* hot spots were included in the study.

### 2.3. Statistical Analyses

Pearson χ^2^ or 2-sided Fisher exact test was used for comparison of categorical variables, and analysis of variance and Kruskal–Wallis test were used for comparison of continuous variables after testing for normality by Shapiro–Wilk test. Survivals were estimated using univariable and multivariable Cox regression analyses and the Kaplan–Meier method. Differences between groups were compared using the log rank test. Statistical significance was set at *p* < 0.05. Data were analyzed using the Statistical Package for the Social Sciences v25 software (IBM Corp., Armonk, NY, USA).

## 3. Results

Among 795 patients, defined as the complete cohort, immunohistochemistry confirmed intact MMR protein expression in 508 (63.9%) and MMR deficiency in 287 (36.1%) (Table 1). Comprehensive molecular characterization was successful for 515 tumors, defined as the “TCGA cohort”. Of these tumors, 218 (42.3%) were classified as NSMP, 191 (37.1%) as MMR-D, 69 (13.4%) as p53 abnormal (abn), and 37 (7.2%) as *POLE* EDM. Twenty cases (3.9%) displayed multiple molecular features. Four cases were classified as *POLE* EDM tumors [32]: three displayed *POLE* EDM and either MMR-D or p53 abn, and one had all three molecular alterations. Sixteen cases were classified as MMR-D tumors [33], displaying both MMR-D and p53 abn. Median follow-up time was 82 months (range 1–136) for the complete cohort and 81 months (range 1–136) for the TCGA cohort.

**Figure 1:**
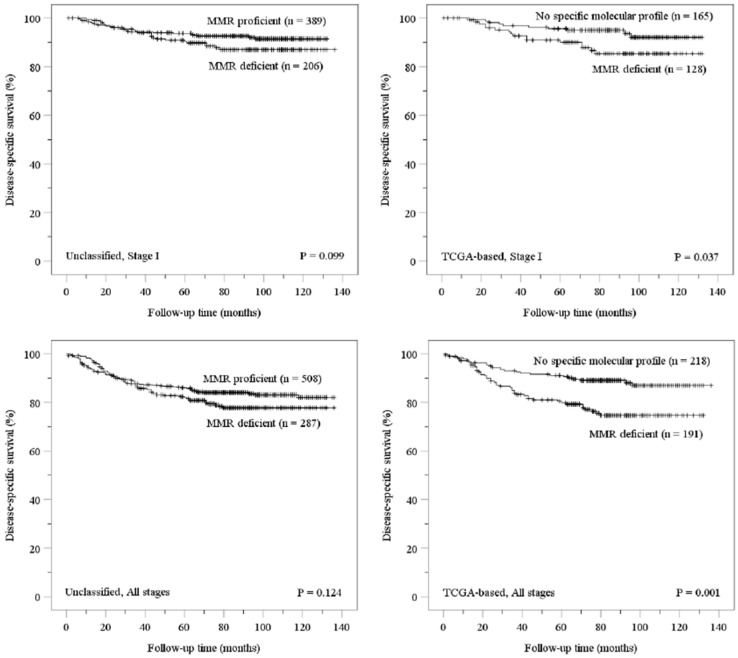
Kaplan–Meier estimations for disease-specific survival in patients with stage I or stage I–IV endometrial carcinoma according to the presence of mismatch repair deficiency. Abbreviations: MMR, mismatch repair; TCGA, The Cancer Genome Atlas.

Kaplan–Meier analyses were performed separately for the complete cohort and the TCGA cohort (Figure 1). Disease-specific survival was similar for MMR proficient and deficient cases in the complete cohort. In contrast, MMR deficiency was associated with poor survival in the TCGA cohort. These findings also applied to tumors that were confined to the uterine corpus (stage I).

Subsequent analyses were performed on the TCGA cohort. Clinicopathologic data for MMR-D and NSMP subgroups are shown in Table 2. Baseline characteristics were balanced between subgroups with the exception of older age, lower body mass index, higher rate of pelvic–aortic lymphadenectomy, lower proportion of well-differentiated endometrioid carcinomas, and higher proportion of cervical stromal invasion and lymphovascular space invasion in the MMR-D subgroup.

Univariable Cox regression disease-specific survival analyses are shown in Supplementary Table S1. Disease extent beyond the uterine corpus, high-risk histotype, deep myometrial invasion, large tumor size, lymphovascular space invasion, and positive peritoneal cytology were associated with an increased risk of death in MMR-D and NSMP subgroups. Further, cervical stromal invasion was associated with an increased risk of death in the MMR-D subgroup, and old age and positive L1CAM expression were associated with increased risk of death in the NSMP subgroup.

To assess the independent effect of MMR status on patient outcome, we performed a multivariable Cox regression analysis of disease-specific survival (Table 3). MMR deficiency was independently associated with poor outcome, similarly to endometrioid grade 3 histology, deep myometrial invasion, large tumor size, lymphovascular space invasion, and positive peritoneal cytology.

Hazard ratios for disease-related death in the presence and absence of various clinicopathologic risk factors are shown in Table 4. MMR deficiency was invariably associated with an increased risk of death in the absence of risk factors. In contrast, outcomes for MMR-D and NSMP subgroups did not differ in the presence of these factors.

Lastly, we examined the association of MMR status on types of relapses in stage I endometrial carcinoma (Table 5). Compared with the NSMP subgroup, the proportion of pelvic relapses was higher in the MMR-D subgroup. Lymphatic dissemination, defined as primary lymph node involvement or relapses in regional lymph nodes, was more common in the MMR-D subgroup (Table 6).

## 4. Discussion

Our study shows no association between endometrial cancer-related survival and MMR deficiency, assessed solely by MMR protein immunohistochemistry (Figure 1). However, when MMR proficient carcinomas were defined as those lacking a specific molecular profile, according to a TCGA-based approach, MMR deficiency was associated with poor survival (Figure 1, Table 3).

These findings indicate that a complete classification into molecular subgroups should ideally be performed to appreciate the prognostic significance of MMR status in endometrial carcinoma. A two-tiered classification into MMR proficient and deficient tumors results in the inclusion of the copy-number high and *POLE* ultramutated subgroups, that is, those associated with the respectively poorest and best outcomes [12] in the MMR proficient category. Thus, the prognostic role of MMR proficiency can be distorted by this approach. It is likely that MMR proficient tumors were heterogeneous in earlier studies based on the identification of MMR deficiency alone [5,6,7,8,9,10,11], which could explain the inconsistent findings on the association of MMR status with patient outcome.

Raffone et al. [34] performed a meta-analysis of individual studies [29,35,36,37,38,39] that provided data about prognosis of TCGA-based subgroups in endometrial carcinoma. An MSI hypermutated subgroup showed a 1.5–2-fold increased risk of overall mortality compared with the NSMP subgroup, which became nonsignificant after adjusting for clinicopathologic factors. An MSI hypermutated subgroup independently worsened overall survival in one study [29], whereas the effect was not significant in five studies [35,36,37,38,39]. We assume that diverse study populations and different selection of confounding variables may explain the discrepant results. The generalizability of the present study may be increased by the fact that our patient sample was unselected with regard to factors such as stage, histology, and tumor size, and a comprehensive set of confounders was included in the multivariable model of disease-specific survival (Table 3).

Patients with MMR-D endometrial carcinomas were older, and their tumors more frequently had aggressive features, including high grade and nonendometrioid histology, cervical stromal invasion, and lymphovascular space invasion (Table 2). These differences may not alone explain the poor outcome in the MMR-D subgroup, based on the apparent independent effect of MMR status on survival in multivariable analysis (Table 3). This prompted us to explore the prognostic effect of MMR status in the absence and presence of established clinicopathologic risk factors (Table 4). Importantly, MMR-D was invariably associated with an increased risk of disease-related death in the absence of risk factors, but the risk was similar for MMR-D and NSMP subgroups when such factors were present. Thus, compared with the NSMP subtype carcinomas, MMR-D subtype carcinomas at risk for relapse and poor outcome appear to be less adequately identified by traditional risk factors.

In agreement with a previous study where MMR deficient endometrial carcinomas were more likely to recur in retroperitoneal lymph nodes [10], we observed that MMR-D subtype carcinomas were prone to lymphatic dissemination (Table 6). Stage I MMR-D subtype carcinomas tended to recur in the pelvis (Table 5), which may reflect the relationship between MMR status and response to adjuvant therapy [40]. We have demonstrated that adjuvant therapies currently used in clinical practice are not associated with improved outcome in MMR-D subtype endometrial carcinomas, as opposed to the NSMP subtype for which adjuvant therapies are associated with a reduced risk of cancer-related death [40].

MSI hypermutated cancers have a high mutation rate and increased neoantigen load, which represent a favorable feature for the implementation of immunotherapy [41]. Thus, immunotherapy is an obvious alternative to more conventional adjuvant therapies in these types of tumors. Clinical benefit of treatment with pembrolizumab, an immune checkpoint inhibitor, in previously treated unresectable or metastatic MSI hypermutated non-colorectal cancers has been demonstrated, with an objective response rate of 34.3% [42].

Our study has some limitations, including its retrospective nature and incomplete molecular characterization of 35% of the primary tumor samples. However, the work is strengthened by detailed clinicopathologic annotation and long follow-up time with cancer-related mortality rather than overall mortality as the outcome of interest.

## 5. Conclusions

The effect of MMR deficiency on the outcome of endometrial carcinoma depends on how MMR proficiency is defined. The poor outcome of MMR deficient carcinomas in the absence of established risk factors emphasizes the need for improved risk stratification of this disease subtype. The observed tendency for pelvic/lymphatic spread of MMR deficient carcinomas may need to be addressed in the design of adjuvant therapy trials.

## Figures and Tables

**Table 1:** Characteristics of the complete cohort (*n* = 795).

Age (Years) (Median (Interquartile Range))	68 (60–75)
Body mass index (kg/m^2^) (edian (interquartile range))	27.3 (23.7–32.4)
Pelvic lymphadenectomy	447 (56.2%)
Pelvic-aortic lymphadenectomy	118 (14.8%)
Stage	
IA	431 (54.2%)
IB	164 (20.6%)
II	54 (6.8%)
IIIA	39 (4.9%)
IIIB	7 (0.9%)
IIIC1	46 (5.8%)
IIIC2	24 (3.0%)
IVA	0 (0%)
IVB	30 (3.8%)
Mismatch repair deficiency	287 (36.1%)
Histology	
Endometrioid carcinoma	702 (88.3%)
Clear cell carcinoma	32 (4.0%)
Serous carcinoma	29 (3.6%)
Carcinosarcoma	17 (2.1%)
Undifferentiated carcinoma	14 (1.8%)
Neuroendocrine carcinoma	1 (0.1%)
Grade (For endometrioid only, *n* = 702)	
1	402 (57.3%)
2	193 (27.5%)
3	107 (15.2%)
Aberrant p53 ^1^	134 (17.1%)
*Polymerase*-*ϵ* ultramutated ^2^	33 (6.4%)
Adjuvant therapy	
Vaginal brachytherapy	383 (48.2%)
Whole pelvic radiotherapy	115 (14.5%)
Chemotherapy	34 (4.3%)
Chemotherapy and vaginal brachytherapy	50 (6.3%)
Chemotherapy and whole pelvic radiotherapy	100 (12.6%)

^1^ Data missing for 11 patients; ^2^ data missing for 277 patients.

**Table 2:** Characteristics of the study population according to subgroups based on The Cancer Genome Atlas.

Variable	MMR-D (*n* = 191)	NSMP (*n* = 218)	*p*
Age (years) (median (interquartile range))	70 (61–77)	66 (60–73)	0.003
Body mass index (kg/m^2^) (median (interquartile range))	27.1 (23.3–32.7)	28.5 (24.3–33.2)	0.042
Pelvic lymphadenectomy	106 (55.5%)	129 (59.2%)	0.453
Pelvic-aortic lymphadenectomy	34 (17.8%)	19 (8.7%)	0.006
Stage			0.077
IA	84 (44.0%)	123 (56.4%)	
IB	44 (23.0%)	42 (19.3%)	
II	19 (9.9%)	23 (10.6%)	
IIIA	13 (6.8%)	9 (4.1%)	
IIIB	2 (1.0%)	1 (0.5%)	
IIIC1	18 (9.4%)	13 (6.0%)	
IIIC2	7 (3.7%)	1 (0.5%)	
IVA	0 (0%)	0 (0%)	
IVB	4 (2.1%)	6 (2.8%)	
Histology			<0.001
Endometrioid grade 1–2	133 (69.6%)	193 (88.5%)	
Endometrioid grade 3	41 (21.5%)	13 (6.0%)	
Nonendometrioid	17 (8.9%) ^1^	12 (5.5%) ^2^	
Myometrial invasion ≥ 50%	89 (46.6%)	83 (38.1%)	0.081
Cervical stromal invasion	42 (22.1%) ^3^	31 (14.2%)	0.038
Tumor size > 5 cm	49 (27.4%) ^4^	44 (21.8%) ^5^	0.205
Lymphovascular space invasion	62 (32.5%)	49 (22.5%)	0.023
Positive peritoneal cytology	10 (5.3%) ^6^	11 (5.1%) ^7^	0.917
L1 cell adhesion molecule	17 (8.9%) ^3^	14 (6.7%) ^8^	
Adjuvant therapy			0.081
Vaginal brachytherapy	83 (43.5%)	116 (53.2%)	
Whole pelvic radiotherapy	35 (18.3%)	28 (12.8%)	
Chemotherapy	8 (4.2%)	7 (3.2%)	
Chemotherapy and vaginal brachytherapy	10 (5.2%)	10 (4.6%)	
Chemotherapy and whole pelvic radiotherapy	35 (18.3%)	24 (11.0%)	

Abbreviations: MMR-D, mismatch repair deficient; NSMP, no specific molecular profile.^1^ Clear cell, *n* = 5; serous, *n* = 3; undifferentiated, *n* = 6; carcinosarcoma, *n* = 3; ^2^ clear cell, *n* = 5; serous, *n* = 2; undifferentiated, *n* = 3; carcinosarcoma, *n* = 2; ^3^ data missing for 1 patient; ^4^ data missing for 12 patients; ^5^ data missing for 16 patients; ^6^ data missing for 4 patients; ^7^ data missing for 3 patients; ^8^ data missing for 8 patients.

**Table 3:** Multivariable Cox regression disease-specific survival analysis (*n* = 364).

Variable	*n*	HR (95% CI)	*p*
Mismatch repair deficiency	173	2.0 (1.1–3.6)	0.024
Age (continuous variable)	364	1.0 (0.98–1.0)	0.635
Stage II–IV	104	2.0 (0.71–5.6)	0.188
Histology			0.111
Endometrioid grade 1–2	289	1	
Endometrioid grade 3	48	2.0 (1.0–3.9)	0.041
Nonendometrioid	27	1.6 (0.72–3.7)	0.239
Myometrial invasion ≥ 50%	148	2.2 (1.1–4.4)	0.033
Cervical stromal invasion	67	0.59 (0.28–1.2)	0.162
Tumor size > 5 cm	91	1.8 (1.0–3.3)	0.047
Lymphovascular space invasion	99	2.6 (1.4–4.7)	0.001
Positive peritoneal cytology	19	4.3 (2.1–9.0)	<0.001
Positive L1 cell adhesion molecule	28	1.5 (0.69–3.3)	0.306
Adjuvant therapy			0.882
None	48	1	
Vaginal brachytherapy	175	0.60 (0.17–2.1)	0.421
Whole pelvic radiotherapy	57	0.72 (0.22–2.4)	0.591
Chemotherapy ± VBT/WPRT	84	0.81 (0.25–2.6)	0.719

Abbreviations: CI, confidence interval; HR, hazard ratio; VBT, vaginal brachytherapy; WPRT, whole pelvic radiotherapy.

**Table 4:** Univariable Cox regression disease-specific survival analyses for endometrial carcinomas according to subgroups based on The Cancer Genome Atlas.

Variable	N MMR-D (*n* = 191)	N NSMP (*n* = 218)	HR (95% CI) (ref: NSMP)	*p*
Age ≤ 65 years	70 (36.6%)	102 (46.8%)	2.9 (1.2–6.8)	0.015
Age > 65 years	121 (63.4%)	116 (53.2%)	1.8 (1.0–3.3)	0.051
Low-risk histology ^1^	133 (69.6%)	193 (88.5%)	2.8 (1.4–5.6)	0.003
High-risk histology ^2^	58 (30.4%)	25 (11.5%)	0.70 (0.35–1.4)	0.332
Myometrial invasion < 50%	102 (53.4%)	135 (61.9%)	2.8 (1.0–7.4)	0.041
Myometrial invasion ≥ 50	89 (46.6%)	83 (38.1%)	1.8 (0.99–3.1)	0.053
Cervical stromal invasion −	148 (77.9%) ^3^	187 (85.8%)	2.0 (1.1–3.5)	0.024
Cervical stromal invasion +	42 (22.1%)	31 (14.2%)	2.3 (0.92–6.0)	0.073
Tumor size ≤ 5 cm	130 (72.6%) ^4^	158 (78.2%) ^5^	2.3 (1.1–4.5)	0.020
Tumor size > 5 cm	49 (27.4%)	44 (21.8%)	1.7 (0.80–3.5)	0.170
Lymphovascular space invasion −	129 (67.5%)	169 (77.5%)	2.2 (1.0–4.9)	0.046
Lymphovascular space invasion +	62 (32.5%)	49 (22.5%)	1.7 (0.89–3.1)	0.108
Peritoneal cytology −	177 (94.7%) ^6^	204 (94.9%) ^7^	3.1 (1.7–5.7)	<0.001
Peritoneal cytology +	10 (5.3%)	11 (5.1%)	0.73 (0.25–2.1)	0.567
L1 cell adhesion molecule −	173 (91.1%) ^3^	196 (93.3%) ^8^	2.9 (1.6–5.1)	<0.001
L1 cell adhesion molecule +	17 (8.9%)	14 (6.7%)	0.76 (0.25–2.4)	0.639

Abbreviations: CI, confidence interval; HR, hazard ratio; MMR-D, mismatch repair deficient; NSMP, no specific molecular profile. ^1^ Grade 1–2 endometrioid carcinoma; ^2^ grade 3 endometrioid and nonendometrioid carcinoma; ^3^ data missing for 1 patient; ^4^ data missing for 12 patients; ^5^ data missing for 16 patients; ^6^ data missing for 4 patients; ^7^ data missing for 3 patients; ^8^ data missing for 8 patients.

**Table 5:** Relapse types in stage I MMR-D and NSMP endometrial carcinomas.

Relapse Type	MMR-D (*n* = 128)	NSMP (*n* = 165)	*p*
Vaginal	0 (0%)	4 (2.4%)	0.134
Pelvic	11 (8.6%)	4 (2.4%)	0.029
Other intra-abdominal	3 (2.3%)	7 (4.2%)	0.522
Extra-abdominal	5 (3.9%)	3 (1.8%)	0.303

Abbreviations: MMR-D, mismatch repair deficient; NSMP, no specific molecular profile. Fisher exact test, 2-sided.

**Table 6:** Occurrence of primary lymph node involvement and recurrences in regional lymph nodes in MMR-D and NSMP endometrial carcinomas.

Type of Lymph Node Metastasis or Relapse	MMR-D (*n* = 191)	NSMP (*n* = 218)	*p*
Stage IIIC1	18 (9.4%)	13 (6.0%)	
Stage IIIC2	7 (3.7%)	1 (0.5%)	
Stage IV with lymph node involvement	1 (0.5%)	1 (0.5%)	
Stage IV with lymph node relapse	3 (1.6%)	1 (0.5%)	
Stage I–IIIB with pelvic lymph node relapse	5 (2.6%)	3 (1.4%)	
Stage I–IIIB with para-aortic lymph node relapse	2 (1.0%)	3 (1.4%)	
Stage I–IIIB with pelvic-aortic lymph node relapse	2 (1.0%)	1 (0.5%)	
Combined	38 (19.9%)	23 (10.6%)	0.008

Abbreviations: MMR-D, mismatch repair deficient; NSMP, no specific molecular profile. Pearson χ^2^ test.

## Data Availability

All relevant data are within the paper.

## Supplementary Materials

The following are available online https://www.mdpi.com/article/10.3390/cancers13133124/s1, Table S1: Univariable Cox regression disease-specific survival analyses for MMR-D and NSMP endometrial carcinomas.
