# Supplementary material for: Mismatch Repair Deficiency as a Predictive and Prognostic Biomarker in Molecularly Classified Endometrial Carcinoma
Source: Cancers (Basel). 2021 Jun 22;13(13):3124. doi: 10.3390/cancers13133124 (PMC8268938; doi:10.3390/cancers13133124)
Supplement: Supplementary file 1 [file cancers-13-03124-s001.zip › cancers-1259917-supplementary.pdf]

Table S1. Univariable Cox regression disease-specific survival analyses for MMR-D and NSMP endometrial carcinomas.

**Variable**	**MMR-D (n = 191)**			**NSMP (n = 218)**		
	**N**	**HR (95% CI)**	**P**	**N**	**HR (95% CI)**	***p***
Age (continuous variable)	191	1.0 (0.98–1.0)	0.835	218	1.1 (1.0–1.1)	0.025
Stage II–IV	63	4.4 (2.4–8.1)	<0.001	53	5.3 (2.4–12)	<0.001
Histology			0.005			<0.001
Endometrioid grade 1-2	133	1		193	1	
Endometrioid grade 3	41	2.7 (1.4–5.2)	0.003	13	14 (5.6–33)	<0.001
Nonendometrioid	17	2.5 (1.0–6.2)	0.046	12	6.6 (2.2–20)	0.001
Myometrial invasion ≥50%	89	3.7 (1.9–7.1)	<0.001	83	5.7 (2.3–14)	<0.001
Cervical stromal invasion	42 [1]	2.6 (1.4–4.8)	0.002	31	2.1 (0.83–5.2)	0.121
Tumor size >5 cm	49 [2]	3.0 (1.6–5.5)	0.001	44 [3]	3.8 (1.7–8.4)	0.001
Lymphovascular space invasion	62	4.6 (2.5–8.4)	<0.001	49	5.9 (2.7–13)	<0.001
Positive peritoneal cytology	10 [4]	4.9 (2.0–12)	<0.001	11 [5]	21 (8.9–48)	<0.001
L1 cell adhesion molecule	17 [1]	1.9 (0.81–4.5)	0.138	14 [6]	7.1 (2.8–18)	<0.001

Abbreviations: CI, confidence interval; HR, hazard ratio; MMR-D, mismatch repair deficient; NSMP, no specific molecular profile.[1] Data missing for 1 patient; [2] data missing for 12 patients; [3] data missing for 16 patients; [4] data missing for 4 patients; [5] data missing for 3 patients; data missing for 8 patients.
